# L3-L4/L4-L5 Type II-A spondylolisthesis: A case report

**DOI:** 10.1016/j.ijscr.2024.110410

**Published:** 2024-10-09

**Authors:** Jonathan McKeeman, Emily Zielinski, Flynn A. Rowan

**Affiliations:** a Department of Orthopaedic Surgery, St. Luke's University Health Network, Bethlehem, PA, United States of America; b Department of Orthopaedic Surgery, Indiana University, Indianapolis, IN, United States of America

**Keywords:** Double level isthmic spondylolisthesis, Gill laminectomy, Posterior instrumented fusion, Transforaminal interbody fusion

## Abstract

**Introduction and importance:**

Double level isthmic spondylolisthesis at L3-L4/L4-L5 is exceedingly rare with only a few documented cases in the literature, but to our knowledge no detailed case reports have been written.

**Case presentation:**

49 year old male with L3–4, L4–5 isthmic spondylolisthesis with neurologic symptoms and failed conservative management treated with L3–4, L4–5 Gill laminectomy, transforaminal interbody fusion with bone grafting and L3–5 posterior instrumented fusion.

**Clinical discussion:**

While rare, this condition can be successfully treated with posterior decompression and instrumented interbody fusion similar to single level spondylolisthesis. Surgeons should feel confident that they can achieve a good outcome for patients and feel comfortable offering this procedure.

**Conclusion:**

This case report may offer guidance for surgeons in the future as it explores the successful treatment of double level isthmic spondylolisthesis at L3-L4/L4–5 from initial presentation to final post-operative follow-up where the patient had complete resolution of symptoms.

## Highlights

• Double level isthmic spondylolisthesis at L3-L4/L4-L5 is exceedingly rare.
• There is no established standard treatment.
• Posterior decompression and instrumented interbody fusion is valid.
• Treatment is similar to a single level spondylolisthesis.

## 1 Introduction

The progression of spondylolysis to isthmic spondylolisthesis is an established, although uncommon, occurrence thought to exist as a continuum of injury that begins with a stress fracture of the pars interarticularis and progresses to slippage of the affected vertebral body in relation to the adjacent caudal vertebral body [1]. Spondylolisthesis can be classified into five basic types: dysplastic (Type I), isthmic (Type II), degenerative (Type III), traumatic (Type IV), and neoplastic (Type V).^1^ Type II isthmic spondylolisthesis is subdivided into types A, B, and C: pars fatigue fracture, pars elongation due to multiple healed stress fractures, and acute pars fracture, respectively [1]. Multiple level spondylolysis is rare, accounting for around 6 % of cases, and even rarer is multiple level isthmic spondylolisthesis [2,3]. Lumbar spondylolisthesis occurs in approximately 4–6 % of the population, and 95 % of cases involve only a single level; most commonly L4-L5 [4]. In a review by Zhang et al., in a cohort of 1700 patients with spondylolisthesis, 24 were found to have multiple level spondylolisthesis, and of these 24 patients, only 9 had multiple level spondylolytic spondylolisthesis [4]. In another study by Song et al., 32 patients were noted to have double-level isthmic spondylolisthesis, with 30 of the cases occurring at L4-L5/L5-S1 and 2 cases occurring at L3-L4/L4-L5 [5]. While these cases have been documented, none appear to be in great detail focusing on the treatment of a patient from initial presentation to final follow up. More recent literature has focused on treatment outcomes for double level spondylolisthesis. Single and double level transforaminal lumbar interbody fusion (TLIF) has been described with significant improvement in patient reported outcome measures (PROMs) [6,7]. Anterior lumbar interbody fusion (ALIF) has also been described with improvement in PROMs [8]. More common appears to be posterior lumbar interbody fusion with a cage and only a few articles mention specifically treating for isthmic spondylolisthesis [5,9]. While it has been described in literature, double level isthmic spondylolisthesis at L3-L4/L4-L5 is exceedingly rare with only a few documented cases and this report seeks to expand upon the literature by presenting a case beginning at initial presentation and through final follow up over one year. This case and work thereafter has been reported in accordance with SCARE criteria [10].

## 2 Clinical case

A 49-year-old male presented to clinic in April 2019 with recurrent low back pain radiating into the bilateral lower extremities, right greater than left. Of note, the patient did have a history of L4/L5 right sided foraminal decompression in 2015. The patient's symptom recurrence began in fall/winter of 2018 with no known traumatic injury. Prior to surgical evaluation, the patient had tried neuropathic medication, formal physical therapy, chiropractics, TENS unit, and deep massage with only minimal improvement in his symptoms.

He was employed as a truck driver, drinks alcohol 1–2 times per week, and never smoked. Chronic problems included non-insulin dependent diabetes mellitus, obesity, hypercholesterolemia, low back pain, and vitamin D deficiency.

**Fig. 1:**
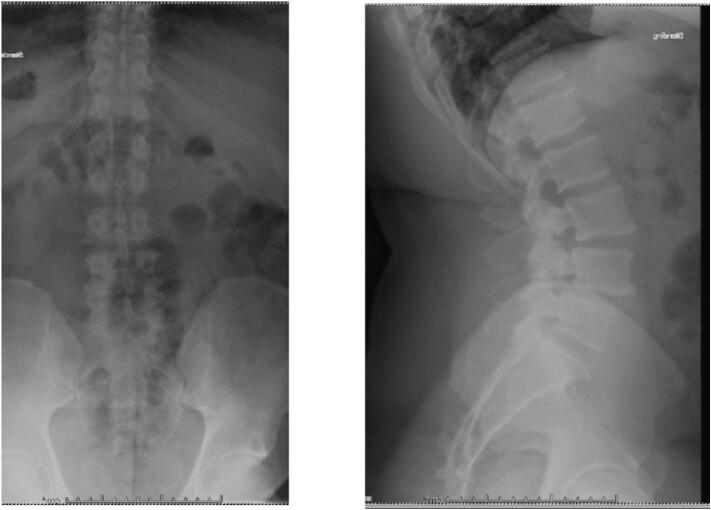
4/10/19 Preoperative Anterior/Posterior and Lateral Radiographs demonstrating L3–4, L4–5 isthmic spondylolisthesis.

**Fig. 2:**
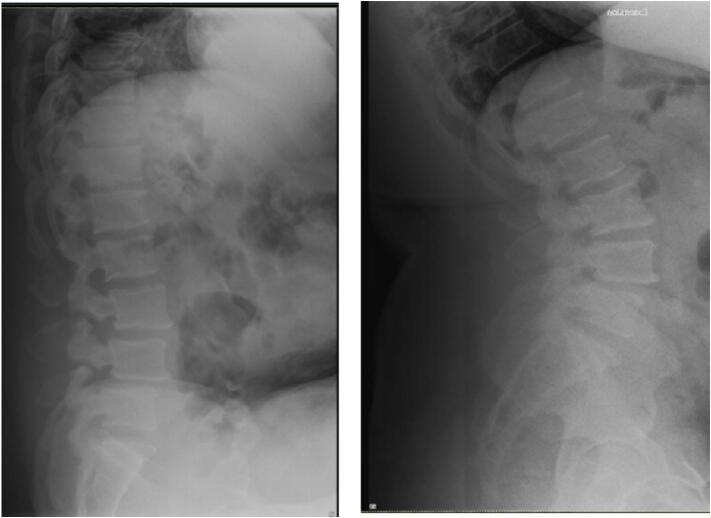
4/10/19 Preoperative Flexion/Extension Lateral Radiographs demonstrating L3–4, L4–5 isthmic spondylolisthesis.

X-rays obtained in clinic demonstrated L3/L4 and L4/L5 spondylolisthesis secondary to L3 and L4 pars defects Fig. 1, Fig. 2. CT imaging confirmed grade I spondylolisthesis (<25 %) at both levels. Lumbar MRI showed intervertebral disc degeneration at L3/L4 and L4/L5 along with right sided foraminal stenosis.

Options for management of the patient's symptoms were discussed including continued conservative therapy versus epidural steroid injection versus surgery. The patient initially opted for a right sided L3–4, L4-L5, transforaminal steroid injection that was performed on 6/26/19, resulting in about 20 % improvement. He returned to clinic over a year later after receiving multiple injections with only minimal relief. Surgery was again discussed, and the patient elected to proceed with L3/L4 and L4/L5 laminectomy and transforaminal lumbar interbody fusion and L3–5 posterior instrumented fusion. Surgery was performed on 2/11/2021.

**Fig. 3:**
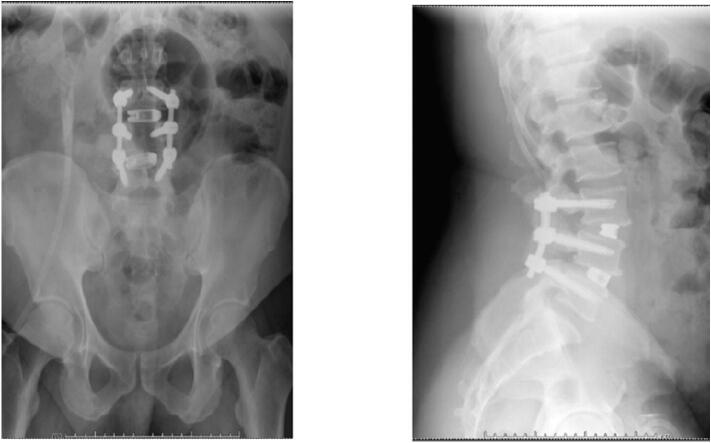
2/12/21- Postoperative Anterior/Posterior and Lateral Radiographs demonstrating intact L3–5 posterior decompression and instrumented fusion with L3–4, L4–5 transforaminal interbody fusion.

**Fig. 4:**
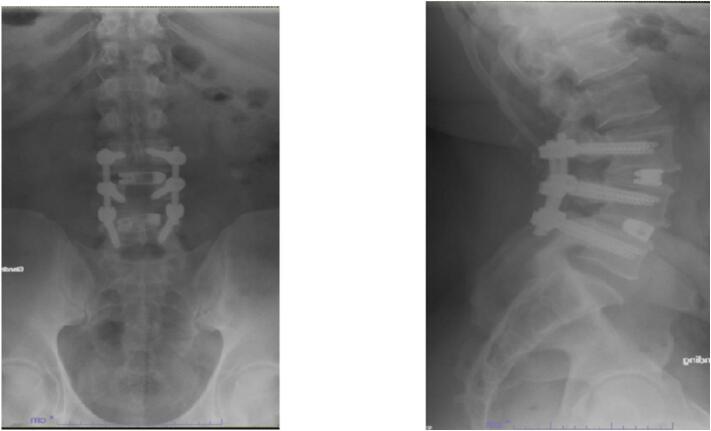
2/1/22- Postoperative Anterior/Posterior and Lateral Radiographs demonstrating intact L3–5 posterior decompression and instrumented fusion with L3–4, L4–5 transforaminal interbody fusion.

At the 2-week post-operative follow up, pain was significantly improved, but the patient endorsed some residual left leg numbness and tingling. Post operative radiographs are shown in Fig. 3. By 3 month follow up, the patient continued to do well. His surgical back pain was improved with only mild midline back soreness. Pre-operative lower extremity symptoms had improved significantly. He was cleared to resume normal activities including bending, lifting, and twisting. He had not yet returned to work as a truck driver at 3 months post-operatively due to ongoing physical therapy. By 6 month follow up he was back at work and tolerating it well, continuing PT, with some mild aching and stiffness. By 1 year follow up he was doing very well and back to all his normal daily activities with less pain and greater function with radiographs shown in Fig. 4. He only reported mild stiffness at this visit. The patient did not return for 2 year follow up.

## 3 Discussion

Since multiple level isthmic spondylolisthesis is so rare, the risk factors and etiology for patients are not certain. The progression of spondylolysis to spondylolisthesis is also not well understood, but there is thought to be a multifactorial origin; genetics, trauma, mechanical, and hormonal factors may contribute [5]. In general, isthmic spondylolisthesis is seen more commonly in males, but double level isthmic spondylolisthesis was three times more common in females in the Song et al. study [5]. .It is difficult to draw conclusions as to why our patient's slip progression occurred and some of the more nebulous factors such as genetics or hormones could play a role, but there were no clear risk factors in this case.

In the review of 32 cases of double level isthmic spondylolisthesis, Song et al. found only 2 cases of L3-L4, L4-L5 spondylolisthesis. The other 30 cases were L4-L5, L5-S1, which is much more common in the already rare double level isthmic spondylolisthesis. In this particular case, the patient had the even rarer double level isthmic spondylolisthesis at the L3-L4, L4-L5 level, which makes it difficult to compare to the majority of cases in the Song et al. study which were L4-L5, L5-S1. However, double level isthmic spondylolisthesis in general can provide an adequate comparison.

The surgical technique for both this case and Song et al. were the same standard posterior midline technique and interbody fusion. The main difference was the addition of Vivigen bone graft to the autologous bone graft procured from the decompression and the technique for the interbody fusion being a TLIF in our case and PLIF for Song et al. The symptoms documents prior to surgery were low back pain, radiating leg pain, neurogenic claudication, numbness, and weakness [5]. Our patient's main symptoms were low back pain and radiating leg pain, the two most common in the Song et al. study at 100 % and 78.1 % respectively [5]. To quantify pain, Song et al. used the VAS pain score preoperatively and post-operatively. For this case, pain was simply assessed as mild, moderate, severe in terms of ability to function and manage activities of daily living. At 2 weeks post-op the patient had already improved to a moderate amount of pain from severe before surgery. Some residual numbness and tingling in his legs remained. Then at his 3 months post-op visit he was feeling much improved and very satisfied with his progress, with complete resolution of neurological symptoms and reduction in pain to mild aching and soreness. This trajectory is similar to that of the patients in Song et al. who reported a VAS pain score average of 6.48 points pre-operatively and then 3.46 points 1 month post-operatively and 2.36 points at 6 months post-operatively [5].

The main radiological result was solid union and reduction in listhesis. As shown in the post-operative radiographs there is a solid union and reduction in anterolisthesis for this patient at both the 2 week follow-up and 3 month follow-up. 87.5 % achieved solid union in the Song et al. study and “the mean degree of listhesis (%)at L3-4,L4-5 and L5-S1 were 7.4%,19.1% and 8.5% respectively; they were changed to 3.2%,3,6% and 2.9%.” [5] In general, patients do very well with this surgery and are overall, very satisfied with their results. Song et al. also report excellent PROMs in their second study and 95 % union rate for the same technique [9].

Several studies have reported on multi-level spondylolisthesis but typically do not specify the type of spondylolisthesis and only a few mention any preoperative workup. This case presents a more detailed report of patient treatment from beginning to end and utilizes a slightly different technique than others reported.

## 4 Conclusion

**Fig. 5:**
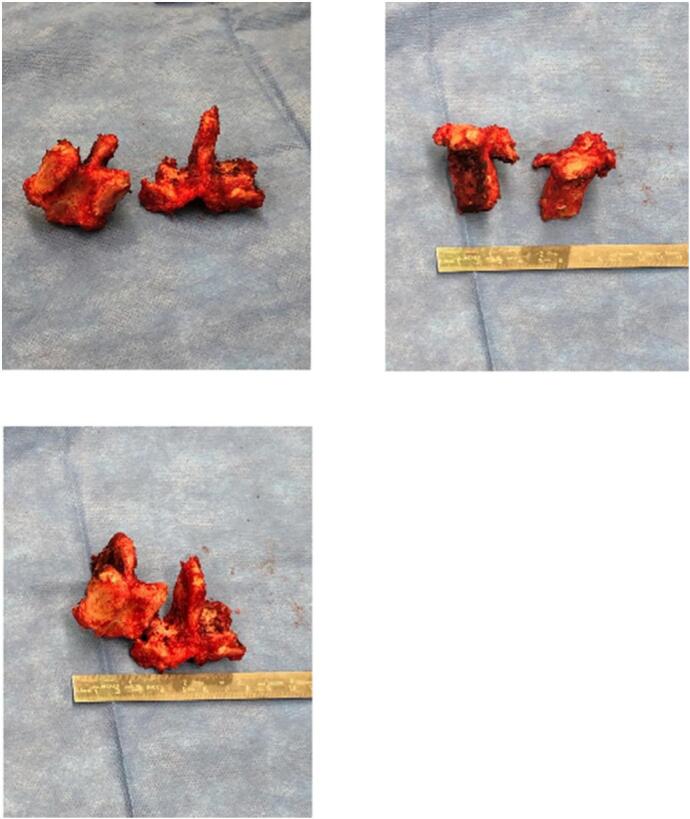
Intra-operative Gill Laminectomy specimens.

Overall, the patient did very well with the Gill laminectomy decompression (Fig. 5) and posterior instrumented fusion and TLIF with bone graft. L3–4/L4–5 isthmic spondylolisthesis is exceedingly rare. This case report may offer guidance for surgeons in the future as it explores the successful treatment of double level isthmic spondylolisthesis at L3-L4/L4–5 from initial presentation to final post-operative follow-up where the patient had complete resolution of symptoms. While this pathology is recognized, our case is unique from others in that it utilizes a TLIF and posterior instrumented fusion from the posterior approach as well as synthetic and autologous bone grafting materials to supplement the fusion construct, which would be our recommendation for surgeons treating this pathology.

## Statement of informed consent

Written informed consent was obtained from the patient for publication of this case report and accompanying images. A copy of the written consent is available for review by the Editor-in-Chief of this journal on request.

## Summary

This is a report of successful treatment of double isthmic spondylolisthesis at L3-L4/L4-L5 that may offer guidance to surgeons encountering this rare condition.

## Ethical approval

The authors are accountable for all aspects of the work in ensuring that questions related to the accuracy or integrity of any part of the work are appropriately investigated and resolved. This study was IRB exempt at the institution of record.

## Funding

No sources of funding were used for this project.

## Author contribution

(I) Conception and design: All Authors.

(II) Administrative support: Flynn A. Rowan MD.

(III) Provision of study materials or patients: Flynn A. Rowan MD.

(IV) Collection and assembly of data: Jonathan McKeeman MD/MBA, Emily Zielinski MD.

(V) Data analysis and interpretation: Jonathan McKeeman MD/MBA, Emily Zielinski MD.

(VI) Manuscript writing: All authors.

(VII) Final approval of manuscript: All authors.

## Guarantor

Jonathan McKeeman MD/MBA.

## Research registration number

N/A.

## Conflict of interest statement

The authors report no disclosures or conflicts of interest.
